# A distinct metabolic signature predicts development of fasting plasma glucose

**DOI:** 10.1186/2043-9113-2-3

**Published:** 2012-02-02

**Authors:** Manuela Hische, Abdelhalim Larhlimi, Franziska Schwarz, Antje Fischer-Rosinský, Thomas Bobbert, Anke Assmann, Gareth S Catchpole, Andreas FH Pfeiffer, Lothar Willmitzer, Joachim Selbig, Joachim Spranger

**Affiliations:** 1 Clinic of Endocrinology, Diabetes and Nutrition, Charité-Universitätsmedizin Berlin, Charitéplatz 1, 10117 Berlin, Germany; 2 Department of Bioinformatics, Institute for Biochemistry and Biology, University of Potsdam, Karl-Liebknecht-Str. 24-25, 14476 Potsdam, Germany; 3 Max-Planck-Institute for Molecular Plant Physiology, Am Mühlenberg 1, 14476 Potsdam, Germany; 4 Department of Clinical Nutrition, German Institute of Human Nutrition, Arthur-Scheunert-Allee 144-116, 14558 Nuthetal, Germany; 5 King Abdulaziz University, P.O. Box 80203 Jeddah 21589, KSA; 6 Experimental and Clinical Research Center (ECRC), Charité-University Medicine Berlin and Max-Delbrück Centre Berlin-Buch, Berlin, Germany; 7 Center for Cardiovascular Research (CCR), Charité-University Medicine Berlin, Berlin, Germany

**Keywords:** prediction, fasting glucose, type 2 diabetes, metabolomics, plasma, random forest, metabolite, regression, biomarker

## Abstract

**Background:**

High blood glucose and diabetes are amongst the conditions causing the greatest losses in years of healthy life worldwide. Therefore, numerous studies aim to identify reliable risk markers for development of impaired glucose metabolism and type 2 diabetes. However, the molecular basis of impaired glucose metabolism is so far insufficiently understood. The development of so called 'omics' approaches in the recent years promises to identify molecular markers and to further understand the molecular basis of impaired glucose metabolism and type 2 diabetes. Although univariate statistical approaches are often applied, we demonstrate here that the application of multivariate statistical approaches is highly recommended to fully capture the complexity of data gained using high-throughput methods.

**Methods:**

We took blood plasma samples from 172 subjects who participated in the prospective Metabolic Syndrome Berlin Potsdam follow-up study (MESY-BEPO Follow-up). We analysed these samples using Gas Chromatography coupled with Mass Spectrometry (GC-MS), and measured 286 metabolites. Furthermore, fasting glucose levels were measured using standard methods at baseline, and after an average of six years. We did correlation analysis and built linear regression models as well as Random Forest regression models to identify metabolites that predict the development of fasting glucose in our cohort.

**Results:**

We found a metabolic pattern consisting of nine metabolites that predicted fasting glucose development with an accuracy of 0.47 in tenfold cross-validation using Random Forest regression. We also showed that adding established risk markers did not improve the model accuracy. However, external validation is eventually desirable. Although not all metabolites belonging to the final pattern are identified yet, the pattern directs attention to amino acid metabolism, energy metabolism and redox homeostasis.

**Conclusions:**

We demonstrate that metabolites identified using a high-throughput method (GC-MS) perform well in predicting the development of fasting plasma glucose over several years. Notably, not single, but a complex pattern of metabolites propels the prediction and therefore reflects the complexity of the underlying molecular mechanisms. This result could only be captured by application of multivariate statistical approaches. Therefore, we highly recommend the usage of statistical methods that seize the complexity of the information given by high-throughput methods.

## Background

High blood glucose reduces life expectancy worldwide [1], and numerous studies have been performed to identify risk factors of impaired glucose metabolism and type 2 diabetes. Nevertheless, this is a topic that is subject to continuing discussion [2-5]. Established classical markers include: family history of diabetes, markers of adiposity, age and glycemic control itself. In recent years, high-throughput methods have been increasingly applied in clinical research [6-10]. In a recent article Wang *et al. *used a metabolomics approach for diabetes risk assessment [11]. They analysed baseline blood samples from 189 individuals that developed type 2 diabetes during a 12 year follow-up period as well as 189 matched control subjects. Using Liquid Chromatography coupled with Mass Spectrometry (LC-MS), they measured 61 metabolites. Applying paired t-test and McNemar's test, they identified isoleucine, leucine, valine, tyrosine and phenylalanine as being highly associated with future diabetes. We here show that multivariate statistical methods should be applied to account for dependencies within the metabolome. In doing so, we were able to define a complex pattern of metabolites that predicts future development of fasting plasma glucose levels with high accuracy. We also compare the quality of prediction between this metabolic pattern and established risk markers.

## Methods

Fasting plasma samples were taken at baseline and at follow-up after an average of six years in subjects who participated in the prospective follow-up of the Metabolic Syndrome Berlin Potsdam (MESY-BEPO) study [12]. We took the samples under standardised conditions in the morning between 8 and 9 a.m. local time after an overnight fast. All patients gave written informed consent and the study was approved by the local ethical committee.

Fasting plasma glucose levels were measured applying a standard hexokinase assay. Furthermore, we analysed metabolic profiles of baseline fasting plasma samples in a random sub-cohort (n = 172; for characterisation see Table 1) using Gas Chromatography coupled with time-of-flight Mass Spectrometry (GC-MS) according to standard operating procedure [10]. We excluded subjects with type 1 or type 2 diabetes at baseline and subjects treated with oral anti-diabetics or insulin. We measured in total 286 metabolites, some of them are not yet identified. The measurements cover various biochemical classes, such as amino acids, carbohydrates, organic acids, fatty acids and steroids.

**Table 1:** Characterisation of the investigated MESY-BEPO sub-cohort

Clinical Characteristics	Baseline	Follow-up
Age [years]	55.7 ± 11.7	61.5 ± 11.5
Gender [% female]	62.2	
Waist circumference [cm]	93.8 ± 13.8	94.6 ± 17.3
Body mass index [kg/m^2^]	28.6 ± 5.2	29.1 ± 5.3
Fasting glucose [mg]	92.1 ± 11.6	100.5 ± 13.6
Δ*glucose *[*mg*/(*dl *· *a*)]	1.0 ± 2.3	
Time between baseline and follow-up [years]	5.6 ± 0.7	

Characterisation of the investigated MESY-BEPO sub-cohort (n = 172) at baseline and follow-up. Data are presented as mean ± standard deviation.

The chromatographic peaks were picked and identified using the Golm Metabolome Database (GMD) [13] and the R package *TargetSearch *[14]. Since missing values only occurred if metabolite concentration went below detection limit, these values were replaced by a value 0.7 times the minimum measured value. Log-transformation and normalisation of the measured relative intensities were performed according to Lisec *et al. *[15].

To quantify the development of fasting glucose levels we calculated the difference between glucose levels normalised by the elapsed time:

$$\Delta glucose=\frac{\text{glucos}{\text{e}}_{follow-up}-\text{glucos}{\text{e}}_{baseline}}{\text{yea}{\text{r}}_{follow-up}-\text{yea}{\text{r}}_{baseline}}$$

in (*mg/dl*)/*a*. We computed Spearman's rank correlation coefficient and p-values to identify significant correlation between Δ*glucose *and single metabolites with a significance level of *α *= 0.05. Significantly correlating metabolites were used to build linear regression models using the R package *stats *as well as Random Forest regression models [16,17] using the R package *randomForest*. The correlation matrix was drawn using the R package *corrplot*. We also performed a nested feature selection based on the Random Forest importance measure as proposed by Svetnik *et al. *[18]. The importance measure is based on the decrease of accuracy, i.e. the increase of the mean square error (MSE), for the out-of-bag portion for each tree. The MSE was calculated for both the true as well as a permuted predictor variable. The difference between them was calculated for all trees, averaged and normalised and hence resulted in the importance measure. By removing the 50% of metabolites with the smallest importance measure, we iteratively bisected the number of metabolites used to predict Δ*glucose *and thus removed irrelevant metabolites. During this reduction the average Random Forest model accuracy remained stable up to a pattern of nine metabolites. Thus, we employed this number to select metabolites for a Random Forest regression analysis to predict Δ*glucose*. Furthermore, we added established risk markers to the Random Forest regression model. These risk markers were: gender, waist circumference, body mass index (BMI), age and baseline fasting glucose levels.

To evaluate the performance of all the regression models, we calculated the Pearson product-moment correlation coefficient between the real and calculated Δ*glucose*. All feature selection and regression modeling was performed and validated on all samples as well as in the subsets of tenfold cross-validation [19]. To test for reliability the cross-validation including the metabolite selection and regression model building was repeated 100 times. Hence, the given cross-validation accuracies reflect the median of this replication.

## Results

### Fasting glucose development

We observed a median Δ*glucose *of 0.8*mg*/(*dl *· *a*), within the range from -3.7*mg*/(*dl *· *a*) to 8.2*mg*/(*dl *· *a*). The standard deviation was 2.3*mg*/(*dl *· *a*). Compared to the baseline level, 61 subjects had a decreased fasting glucose level (Δ*glucose *≤ 0*mg*/(*dl *· *a*)), whereas 111 subjects showed an increased fasting glucose level (Δ*glucose >*0*mg*/(*dl *· *a*)).

### Correlation

To identify metabolites that are associated with the development of fasting plasma glucose levels, we calculated the Spearman's rank correlation coefficient between the single metabolites and Δ*glucose*. We used the Spearman correlation since not all metabolites were normally distributed. All 30 metabolites that are significantly correlated with Δ*glucose *are shown in Table 2. The observed correlation ranges from -0.27 to 0.40. Therefore, a single metabolite explains up to 16.2% of the observed variance in Δ*glucose *(see Table 2). Consequently, the question arose whether the metabolites are not only capable of explaining but also predicting Δ*glucose*. Thus, we applied a multivariate approach.

**Table 2:** Spearman's rank correlation

	Spearman Correlation	p-value	% variance explained
Hypoxanthine	0.40	*<*0.0001	16.20
Aspartic acid	0.30	0.0001	9.18
Pyroglutamic acid	0.29	0.0001	8.34
2-methyl-Malic acid	0.27	0.0004	7.21
NA 1033 (trisaccharide)	-0.27	0.0004	7.05
NA 1034	-0.23	0.0026	5.23
NA 1052 (carbohydrate)	-0.22	0.0046	4.64
Myo-inositol	0.21	0.0052	4.51
NA 1027 (sterol phosphate)	0.21	0.0061	4.34
Threonic acid	0.21	0.0063	4.31
NA 997 (Uridine-5'-monophosphate)	0.20	0.0078	4.09
Glutamic acid	0.19	0.0104	3.80
NA 831	0.19	0.0119	3.67
NA 653	0.18	0.0179	3.25
Ketopentose	0.18	0.0181	3.24
Fucose	0.18	0.0192	3.18
Uracil	0.18	0.0213	3.08
Fructose	-0.17	0.0233	2.99
NA 631 (D-Glucopyranose)	-0.17	0.0235	2.98
NA 613	0.17	0.0251	2.91
NA 597 (pyranose)	-0.16	0.0312	2.70
Isoleucine	0.16	0.0358	2.57
NA 275	0.16	0.0379	2.51
NA 639	0.16	0.0388	2.49
NA 442	0.16	0.0395	2.47
NA 560	0.16	0.0402	2.45
NA 854 (carbohydrate)	0.16	0.0413	2.43
Tartaric acid	0.15	0.0429	2.39
Fructose	-0.15	0.0466	2.31
NA 632	-0.15	0.0486	2.27

Spearman's rank correlation coefficient, p-values and % of explained variance for all metabolites significantly correlated with Δ*glucose *(significance level *α *= 0.05). Not yet identified metabolites are marked with NA, putative biochemical structures are given in round brackets.

### Linear Model

**Figure 1:**
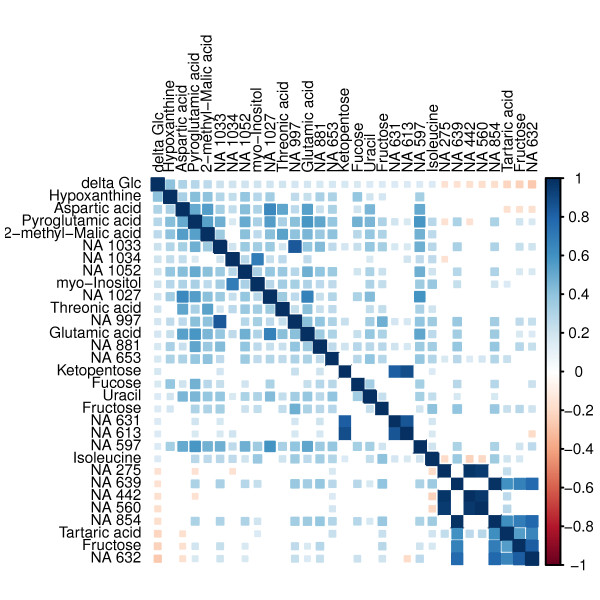
**Correlation matrix**. This correlation matrix visualises not only the significant correlations between Δ *glucose *and the metabolites (first row/column) but also the correlation among the metabolites. The colour intensity and tile size indicate the strength of correlation. Positive correlation are marked blue, negative correlation are marked red.

We used the Spearman correlation as a filter and included all metabolites significantly correlated with Δ*glucose *to build a linear regression model. The accuracy (i.e. the Pearson correlation between true and estimated Δ*glucose*) using all samples was 0.57, the median accuracy after tenfold cross-validation was 0.22. This accuracy is unsatisfactorily poor. Despite the weak accuracy of the linear model, it is known that multicollinearity often leads to an unstable model and affects the correct calculation of coefficients of linear regression models [20]. The calculated condition number of the correlation matrix *K > >*1000 indicated the strong collinearity within the chosen metabolites. However, high correlation within a metabolite matrix is not surprising, since the observed metabolites are not independent but connected via metabolism. The detected multicollinearity is illustrated with the correlation matrix in Figure 1. Hence, we had to find a regression model that is robust against multicollinearity. This characteristic is given for Random Forest models [21].

### Random Forest Regression Models

We built a Random Forest regression model using the metabolites selected by significant correlation to predict Δ*glucose*. Compared to the linear model, the accuracy increase was noteworthy: using all samples the accuracy was 0.97, and after tenfold cross-validation the median accuracy was 0.41. Nevertheless, we investigated the scale of restriction due to using only metabolites that correlate with Δ*glucose*.

**Figure 2:**
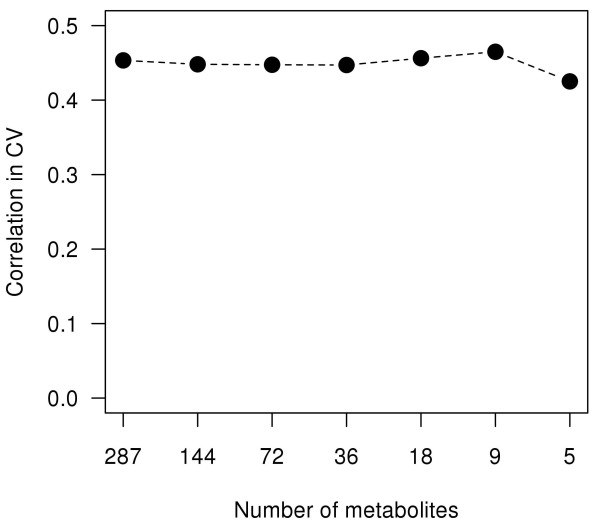
**Random Forest feature selection**. Iterative bisection of the number of metabolites by removing the 50% of metabolites with the smallest importance measure. The remaining metabolites were used to build the Random Forest regression model. Shown is the median cross-validation accuracy. The accuracy remains stable up to a pattern of nine metabolites.

Thus, we performed a nested feature selection based on the Random Forest importance measure. To define a minimum number of metabolites necessary for accurate prediction of Δ*glucose*, we stepwise bisected the number of metabolites. During this reduction the average model accuracy remained stable up to a pattern of nine metabolites (see Figure 2). Therefore, it is legitimate to use only the nine metabolites with the highest importance in the Random Forest model. These nine metabolites are shown in Table 3. The accuracy using these metabolites in a Random Forest regression model was 0.97. The median cross-validation accuracy was 0.47. Although the current selection of metabolites is smaller than the correlation based selection, the accuracy improved. Detailed examination of the two metabolite selections revealed an incomplete overlap. Metabolites highly correlated with Δ*glucose *also showed a high Random Forest importance. However, some metabolites (*e.g*. the putative allantoin, citric acid and an unknown) showed high importance but no significant correlation with Δ*glucose*. We assume that these metabolites are responsible for the increase in accuracy. Therefore, we conclude that not only linear but more complex relations may exist between metabolites and the fasting glucose development. Moreover, this assumption of complexity is underlined by the selected metabolites themselves and their location in biochemical pathways. The identified metabolites of the pattern are part of multiple metabolic pathways, *e.g*. purine degradation, energy metabolism and amino acid metabolism.

**Table 3:** Random Forest Importance of the highest ranked metabolites

Metabolite	Random Forest Importance
Hypoxanthine	12.52
Pyroglutamic acid	9.37
NA 1027 (sterol phosphate)	8.13
NA 611 (Allantoin)	7.80
NA 718 (carboxylic acid)	7.56
NA 1033 (trisaccharide)	6.28
Aspartic acid	5.34
NA 1034	5.26
Citric acid	4.60

Metabolites belonging to the pattern identified using Random Forests and their Importance. Not yet identified metabolites are marked with NA, putative biochemical structures are given in round brackets.

Furthermore, we added established risk markers (gender, waist circumference, BMI, age, baseline fasting glucose) to the metabolites and again performed feature selection based on Random Forest importance and built a Random Forest regression model. Surprisingly, these established risk markers did not improve the accuracy of the model. To further analyse this observation, we built a Random Forest regression model using only the established risk factors. This model had a cross-validation accuracy of 0.05, and therefore was not able to predict Δ*glucose*.

All results of the different regression models can be found in Table 4. For identification of the so far unknown metabolites of our pattern, we revised the original spectra data using the Decision Tree tool provided by Hummel *et al. *[22] and added the information in Table 3.

**Table 4:** Regression model accuracy

Metabolite Selection	Model	all samples	CV
Spearman Correlation	linear Model	0.57	0.22
Spearman Correlation	Random Forest	0.97	0.41
RF importance	Random Forest	0.97	0.47
RF importance + Established markers	Random Forest	0.97	0.46
Established markers	Random Forest	0.90	0.05

Accuracy of the models (median Pearson correlation between real and estimated Δ*glucose *levels) based on metabolites and/or established risk markers was calculated using all samples of the training set and after tenfold cross-validation (CV). The established risk markers are: gender, waist circumference, BMI, age and baseline fasting glucose levels.

## Discussion

In conclusion, our work demonstrates that metabolic profiles have a high performance in predicting fasting glucose level development (Table 4). However, this prediction ability was captured by using non-parametric Random Forest regression. This indicates that a rather complex non-linear pattern allows the prediction of Δ*glucose*.

We also observed that metabolic profiles hold considerably more information than established markers. This may result from the different scopes of these variables: established markers describe a macroscopic phenotype that is underpinned by the molecular level. Although established markers may differ between healthy and diseased subjects, understanding diseases on their molecular level will support the development of individualised medicine and hence lead to better prevention and treatment in clinical practice.

We are aware that diurnal variation or other factors (*e.g*. physical activity or nutrition during the days before phenotyping) might have affected measurements of metabolite and glucose concentrations in our samples. We have tried to minimise this variation by sampling under standardised conditions. Due to the potentially remaining variance, important metabolites might not be included in the model, thus causing false negatives. Consequently, we might have underestimated the capacity of metabolites to predict glycemic control.

To put the metabolites belonging to the final pattern (Table 3) into a biochemical context, we reviewed existing literature.

Among all metabolites hypoxanthine showed the highest importance regarding fasting glucose development. It is a central intermediate in purine degradation and biosynthesis. Hypoxanthine is either enzymatically metabolised via xanthine to uric acid, or re-utilised to inosine monophosphate in the nucleotide salvage pathway. In the past decades several studies have shown an association between the purine degradation pathway and type 2 diabetes or the metabolic syndrome [23]. However, the causality of this association remains unclear. In their recent meta-analysis Pfister *et al. *did not observe a direct effect of serum uric acid or associated genetic variants on development of type 2 diabetes [24]. Nevertheless, other ways of interaction may exist [25,26]. As early as 30 years ago, Harkness *et al. *described that purine degradation is increased under ATP depleting conditions and small changes in hypoxanthine may reflect alterations of ATP turnover [27]. Whereas glucose uptake does not change hypoxanthine levels or purine degradation [28], fructose uptake leads to hepatic ATP degradation [25] and may play a major role in epidemic of metabolic syndrome and obesity [29]. The link between insulin resistance and increased serum urate might be mediated by the hexose phosphate shunt [26]. Elevated hypoxanthine levels might reflect an early stage of this mechanism.

The unknown NA611 is most likely allantoin. This metabolite is also closely related to purine degradation: Although it is not enzymatically produced in human body (due to knock-out mutation in urate oxidase gene [30]), it was referenced before in human blood, cerebrospinal fluid and urine. It is generated by spontaneous reaction of uric acid with radical oxygen species (ROS) [31]. Thus, allantoin levels reflect uric acid action as a ROS scavenger. The role of oxidative stress and ROS in diabetes development has been reviewed elsewhere [32].

Pyroglutamic acid (or 5-oxoproline) is a cyclised derivative of L-glutamic acid. In biological context it can be formed non-enzymatically from glutamate, glutamine and *γ*-glutamylated peptides or enzymatically by *γ*-glutamylcyclotransferase. The latter is part of the *γ*-glutamyl cycle, which synthesises and degrades glutathione. Glutathione is a main intracellular antioxidant [33] and thus plays an important role in maintaining redox homeostasis and eliminating ROS in the cell. S-glutathionylation of proteins as result of redox imbalance is thought to be a biological switch [34]. In our measurements pyroglutamic acid might also be derived from glutamic acid or glutamine during the derivatisation process. Both amino acids act not only in protein synthesis and degradation. Due to their potential to transfer amino groups, they occur in numerous pathways. The activity of several Glx consuming or producing enzymes (*e.g. γ*-glutamyltransferase [35-42], glutamine fructose-6-phosphate amidotransferase [43], glutamate pyruvate transaminase [40]) were reported to be associated with diabetes mellitus, impaired glucose tolerance and insulin resistance and are used as biomarkers to monitor liver functionality. Glutamate links the tricarboxylic acid (TCA) cycle with amino acid biosynthesis and degradation. Furthermore, several animal models were described that develop obesity and/or diabetes after injection of monosodium glutamate [44-48]. In such an animal model reduced nitric oxide and increased ROS production were also observed [49]. In addition, dietary monosodium glutamate increases weight gain, adiposity and reduces insulin sensitivity in an animal model [50]. According to Samocha-Bonett *et al*., oral glutamine reduced postprandial glycemia in type 2 diabetic subjects [51]. Finally, due to its role as neurotransmitter, glutamate is involved in numerous signaling processes [52,53] but is also potentially neurotoxic [54].

Aspartic acid is involved in protein synthesis and degradation and also acts in transamination reactions. It is directly linked to the TCA cycle via the malate shuttle. The aspartate aminotransferase was reported to be associated with glucose metabolism and insulin resistance [38]. Bousova *et al. *reported reduced aspartate amino transferase activity induced by fructose and the resulting glycation *in vitro *and observed beneficial effects of uric acid in that context [55]. Hageman *et al. *reported that feeding an aspartate rich protein blend decreased the postprandial glucose response in rats [56]. According to Arai *et al. *monosodium aspartate induces obesity, increased plasma insulin and increased acetyl-CoA carboxylase in animal models [57].

Citric acid is an intermediate of the TCA cycle, which is a central pathway in the organisms energy preservation. The TCA cycle oxidatively degrades acetyl-CoA, which originates from carbohydrate, amino acid or fatty acid degradation. An animal model for diabetes showed increased gluconeogenesis by increased TCA cycle substrate uptake and fluxes [58]. Citric acid is produced by citrate synthase. Ortenblad *et al. *[59] reported a reduced basal citrate synthase activity, reduced lipid oxidation and reduced insulin-mediated glucose oxidation in culture skeletal muscle cells from type 2 diabetic persons. They increased citrate synthase activity by incubation with insulin in cells from non-diabetic but not in cells from diabetic subjects. Co-incubation with palmitate abolished the stimulatory effect [59]. Furthermore, maximum velocity of citrate synthase was reduced in cultured pancreatic islet cells cultured with long-chain fatty acids or high dose glucose, which may play a role for glucotoxicity and lipotoxicity in *β*-cells [60]. Citrate also connects the TCA cycle with hepatic fatty acid and cholesterol synthesis by being exported via transport proteins from mitochondrion to cytosol where it is cleaved to acetyl-CoA by ATP-citrate lyase. The citrate carrier is the key component of citrate-malate-shuttle and it is located in the mitochondrial inner membrane. High levels of the citrate carrier can be found predominantly in liver, pancreas and kidney [61]. A reduction of citrate carrier activity and protein levels, both affected by insulin and glucose levels at different regulatory steps, was found in diabetic mice [62]. Knockout of gene SLC13A5, encoding for a sodium-coupled plasma membrane citrate transporter, protects mice from adiposity and insulin resistance [63]. The same group reported a 3-fold increased expression of SLC13A3, encoding another hepatic plasma membrane tri/dicarboxylate transporter, in the knockout mice. ATP-citrate lyase is the key enzyme in cellular lipid production. Its activity and mRNA levels are decreased in pancreatic islets of diabetic rats [64]. Administration of insulin increased ATP-citrate lyase activity and mRNA levels in liver of diabetic rats [65]. Chu *et al. *reported a suppression of ATP-citrate lyase expression and activity by palmitate and its critical role in pancreatic *β*-cell survival based on increased *β*-cell apoptosis in knockdown mutation [66]. A liver specific ATP-citrate lyase abrogation improved the systemic glucose metabolism in leptin receptor deficient mice [67]. Moreover, human platelets showed increased ATP-citrate lyase activity in diabetic subjects [68]. Furthermore, a decrease of adipose tissue and plasma insulin concentration were observed in diabetic mice fed with citric acid [69]. In addition, reduced urinary citrate excretion induced by insulin resistance may mediate the development of kidney stones in patients with the metabolic syndrome [70].

Taken together, the metabolites identified so far were reported to be associated with type 2 diabetes and fasting plasma glucose in numerous studies. The metabolites of our pattern seem to be representatives of an early stage of a perturbed energy metabolism. This is reflected by metabolites that are among other pathways closely linked to the TCA cycle as well as by metabolites of the purine degradation. The metabolites linked to the TCA cycle are also involved in many other pathways. Thus, they might be metabolic hubs that transfer the perturbation in energy metabolism to other pathways (*e.g*. amino acid metabolism) or *vice versa*. Subsequently, this perturbation transfer results in the complexity of fasting plasma glucose development. Furthermore, redox homeostasis seems to be involved in this development.

For a complete interpretation of our results the identification of the unknowns is desirable. However, the problem of a high number of unknown metabolites is still an intrinsic problem of metabolite profiling methods. In fact, many studies do not even consider the unknowns to circumvent this problem. However, our study demonstrates the potential importance of such unknowns, even if a complete identification is beyond the scope of this manuscript.

The identified metabolic pattern includes metabolites that are not part of a single pathway, but spread over several pathways. We conclude that glucose level development is comparable to a complex 'clockwork' with multiple key regulators. Small perturbations to single regulators can be buffered by the system, whereas bigger perturbation at several parts disturb the fine tuned balance and lead to elevated blood glucose levels. We interpret the metabolites that are part of the identified metabolic pattern as representatives for the regulatory parts of the 'clockwork'.

This complexity reflects the diabetes research results made on the genome level during the past years: no single gene, but rather a high number of genes are associated with type 2 diabetes [71-73] and fasting glucose development [74].

Finally, this complexity is the challenge for development of individualised medicine. For proper treatment, all disturbed metabolic regulators have to be identified and brought back to normal parameters.

We have mentioned above a recent study investigating the ability of single metabolites measured using LC-MS to predict type 2 diabetes and finally identifying five amino acids [11]. Although their approach is similar in style to ours, there are some differences that make a direct comparison delicate. These differences concern the technical platforms to measure metabolite levels, the number of metabolites measured, the number of patients, observation period, the prediction endpoints and the statistical approaches. Thus, further studies are needed to confirm the results.

Finally, to comment on the practical value of our work: We interpret our results as a first hint that complex metabolite pattern can predict clinical relevant endpoints and thus reveal new insights in molecular mechanisms at early stages of disease development. With respect to diabetes our results need to be confirmed in external cohorts before any application in a clinical setting.

## Conclusions

Taken together, our results indicate that specific metabolites are able to predict development of fasting glucose. However, this prediction is primarily driven by a complex pattern of metabolites rather than single metabolites, demonstrating the interrelation between different metabolic pathways in the regulation of circulating metabolite profiles and in the development of type 2 diabetes.

## Competing interests

The authors declare that they have no competing interests.

## Authors' contributions

MH and AL contributed equally to this manuscript and participated in study design, coordination, statistical analysis, interpretation of the results, discussion and preparation of the manuscript. FS participated in acquisition of the MESY-BEPO data and discussion. AFR participated in acquisition of the MESY-BEPO data and discussion. TB participated in acquisition of the MESY-BEPO data and discussion. AA participated in acquisition of the MESY-BEPO data and discussion. GSC participated in acquisition of GC-MS data and discussion. AFHP participated in acquisition of the MESY-BEPO data and discussion. LW participated in acquisition of GC-MS data and discussion. JSE participated in study design, interpretation of the results, discussion and helped to prepare the manuscript. JSP participated in study design, interpretation of the results, discussion and helped to prepare the manuscript. All authors read and approved the final manuscript.
